# Supplementary material for: Metagenomic sequencing of the skin microbiota of the scalp predicting the risk of surgical site infections following surgery of traumatic brain injury in sub-Saharan Africa
Source: PLoS One. 2024 Jul 24;19(7):e0303483. doi: 10.1371/journal.pone.0303483 (PMC11268656; doi:10.1371/journal.pone.0303483)
Supplement: S1 File — (PDF) [file pone.0303483.s001.pdf]

Uganda National Council for Science and Technology
(Established by Act of Parliament of the Republic of Uganda)

Our Ref: HS1284ES **17 March 2021**

Herve Monka Lekuya
Makerere University
Kampala

Re: Research Approval: **Depressed Skull fractures: Timing of surgery, systemic inflammatory response, intraoperative blood loss, surgical site infection, and post-traumatic seizures - (DESTINE study)**

I am pleased to inform you that on **17/03/2021**, the Uganda National Council for Science and Technology (UNCST) approved the above referenced research project. The Approval of the research project is for the period of **17/03/2021** to **17/03/2022**.

Your research registration number with the UNCST is **HS1284ES**. Please, cite this number in all your future correspondences with UNCST in respect of the above research project. As the Principal Investigator of the research project, you are responsible for fulfilling the following requirements of approval:

1. Keeping all co-investigators informed of the status of the research.

2. Submitting all changes, amendments, and addenda to the research protocol or the consent form (where applicable) to the designated Research Ethics Committee (REC) or Lead Agency for re-review and approval **prior** to the activation of the changes. UNCST must be notified of the approved changes within five working days.

3. For clinical trials, all serious adverse events must be reported promptly to the designated local REC for review with copies to the National Drug Authority and a notification to the UNCST.

4. Unanticipated problems involving risks to research participants or other must be reported promptly to the UNCST. New information that becomes available which could change the risk/benefit ratio must be submitted promptly for UNCST notification after review by the REC.

5. Only approved study procedures are to be implemented. The UNCST may conduct impromptu audits of all study records.

6. An annual progress report and approval letter of continuation from the REC must be submitted electronically to UNCST. Failure to do so may result in termination of the research project.

Please note that this approval includes all study related tools submitted as part of the application as shown below:

No.	Document Title	Language	Version Number	Version Date
1	Informed Consent forms	English + Luganda	2	
2	Data collection tools	English	2	
3	Project Proposal	English	2	
4	Approval Letter	English	2	0000-00-00
5	Administrative Clearance	English	2	0000-00-00

Yours sincerely,

Hellen Opolot
For: Executive Secretary
UGANDA NATIONAL COUNCIL FOR SCIENCE AND TECHNOLOGY

LOCATION/CORRESPONDENCE

Plot 6 Kimera Road, Ntinda
P.O. Box 6884
KAMPALA, UGANDA

COMMUNICATION

TEL: (256) 414 705500
FAX: (256) 414-234579
EMAIL: info@uncst.go.ug
WEBSITE: http://www.uncst.go.ug

TELEPHONE: +256-41554008/1
FAX: +256-414-5325591
E-mail: admin@mulago.or.ug
Website: www.mulago.or.ug

MULAGO NATIONAL REFERRAL HOSPITAL
P. O. Box 7051
KAMPALA, UGANDA

IN ANY CORRESPONDENCE ON THIS SUBJECT PLEASE QUOTES NO……………..

Dr. Nakwagala
Plse handle
12/3/2021

MULAGO NATIONAL REFERRAL HOSPITAL
10 MAR 2021
EXECUTIVE DIRECTOR RECEIVED
MULAGONATIONALREFERRALHOSPITAL

9th March 2021.

The Executive Director
Mulago National Referral Hospital

Dear Sir,

RE: RECOMMENDATION FOR ADMINISTRATIVE CLEARANCE.

The Mulago Hospital Research & Ethics Committee has reviewed the protocol titled MHREC **2049: "Depressed Skull Fractures: Timing of Surgery, Systemic Inflammatory Response, Intraoperative Blood Loss, Surgical Site Infection, and Post Traumatic Epilepsy (DESTINE Study)"** by Dr. Lekuya Monka Herve as the lead Principal Investigator.

The study got approval from Makerere University School of Medicine Research & Ethics Committee for a period of one (1) year from 16th February 2021 to 16th February 2022.

The study has met the following obligations;

1. Paid the MHREC review fees of 100,000/=
2. Agreed to comply with all institutional policies and regulations of Mulago National Referral Hospital
3. Agreed to provide end of study report and acknowledge Mulago hospital in all publications

Administrative clearance was granted for one (1) year on 4th March 2021 valid till 3rd March 2022.

The Investigator should ensure to get final approval of the protocol and all accompanying documents from UNCST before starting the study. In case of studies involving drug approval is obtained from National Drug Authority and for those studies involving medical devices, seek approval from Director General, Ministry of Health.

The study is therefore recommended for your provision of administrative clearance by Mulago National Referral Hospital.

Yours sincerely;

DR. NAKWAGALA FREDERICK NELSON
CHAIRMAN- MULAGO HOSPITAL RESEARCH & ETHICS COMMITTEE.

DR. LEKUYA MONKA
ADM CLEARANCE PROVIDED
15/3/2021

Copied to;

1. **Dr. Lekuya Monka Herve**
2. **Executive secretary – Uganda National Council for Science & Technology.**

MAKERERE UNIVERSITY
P.O. Box 7072 Kampala, Uganda
E-mail: rresearch9@gmail.com
Phone: 256 414 533541
Fax: 256 414 541036/0414 532204
COLLEGE OF HEALTH SCIENCES
SCHOOL OF MEDICINE
RESEARCH ETHICS COMMITTEE

16/02/2021

To: Herve Lekuya

0753158129

Type: Initial Review

Re: SM-2020-7: Depressed Skull fractures: Timing of surgery, systemic inflammatory response, intraoperative blood loss, surgical site infection, and post-traumatic seizures - (DESTINE study), 1, 2020-11-01

I am pleased to inform you that at the **114** convened meeting on **08/01/2021**, the MAK School of Medicine REC (Mak-SOMREC), committee meeting, etc voted to approve the above referenced application.
Approval of the research is for the period of **16/02/2021** to **16/02/2022.**

As Principal Investigator of the research, you are responsible for fulfilling the following requirements of approval:

1. All co-investigators must be kept informed of the status of the research.
2. Changes, amendments, and addenda to the protocol or the consent form must be submitted to the REC for re-review and approval **prior** to the activation of the changes.
3. Reports of unanticipated problems involving risks to participants or any new information which could change the risk benefit: ratio must be submitted to the REC.
4. Only approved consent forms are to be used in the enrollment of participants. All consent forms signed by participants and/or witnesses should be retained on file. The REC may conduct audits of all study records, and consent documentation may be part of such audits.
5. Continuing review application must be submitted to the REC **eight weeks** prior to the expiration date of **16/02/2022** in order to continue the study beyond the approved period. Failure to submit a continuing review application in a timely fashion may result in suspension or termination of the study.
6. The REC application number assigned to the research should be cited in any correspondence with the REC of record.
7. You are required to register the research protocol with the Uganda National Council for Science and Technology (UNCST) for final clearance to undertake the study in Uganda.

The following is the list of all documents approved in this application by MAK School of Medicine REC (Mak-SOMREC):

No.	Document Title	Language	Version Number	Version Date
1	Revised Doctoral Committee	English	1	2020-10-26
2	Informed Consent forms	English + Luganda	1	2020-11-01
3	Protocol	English	1	2020-11-01
4	Data collection tools	English	1	2020-11-01

Yours Sincerely

Ponsiano Ocama
For: MAK School of Medicine REC (Mak-SOMREC)
