# Supplementary material for: Metagenomic sequencing of the skin microbiota of the scalp predicting the risk of surgical site infections following surgery of traumatic brain injury in sub-Saharan Africa
Source: PLoS One. 2024 Jul 24;19(7):e0303483. doi: 10.1371/journal.pone.0303483 (PMC11268656; doi:10.1371/journal.pone.0303483)
Supplement: S2 File — (PDF) [file pone.0303483.s002.pdf]

Author contributions: HML and DPK did the project conceptualization; HML, JPOK, DPK, and MG acquired the funding; HML, LKK, and RN did the project administration and investigations; HML, DPK, GO, and EK did the data curation, formal analysis, software, and visualization; SC, DPK, JV, EB, JPOK, and MG study supervision and validation; HML drafted the original manuscript; DPK, SPM, RM, SC, JV, EB, MG, and JPOK did the critical review and manuscript editing. The final version was approved by all authors.

List of abbreviations: DMM: Dirichlet multinomial machine learning method; DSF: Depressed skull fracture; GCS: Glasgow coma scale; LDA: Linear discriminant analysis; MNRH; Mulago National Referral Hospital; OTU: Operational taxonomic unit; PCoA: Principal component analysis; SSA: sub-Saharan Africa; SSI: Surgical site infection; TBI: Traumatic brain injury.

List and description of tables, figures, and supporting information files.

Table 1: Distribution of patients' baseline demographics and clinical type of injury by outcomes of the Surgical Site Infections.

Table 2: Distribution of microbial culture and antibiotic susceptibility among patients with SSI.

Figure 1: Illustration of normal skin swabbing: (A) Swabbing site at the scalp hairline behind the ear of the non-injured side; (B): Sterile transportation and preservation of the skin swab sample.

Figure 2: Patients' flow chart.

Figure 3: Skin microbiota Phylum relative abundancy by age group.

Figure 4: Skin microbiota absolute abundancy by genera by sex.

Figure 5: Relative abundancy plot of both infected and non-infected groups by genera.

Figure 6: Hierarchical cluster of infected and non-infected samples (Bray).

Figure 7: Clustering tree of individual samples of both infected and non-infected patients by stacked bar plots of phylum.

Figure 8: Absolute abundancy bar plot of both infected and non-infected groups by genera.

Figure 9: Relative abundancy bar plot of both infected and non-infected groups by genera.

Figure 10: Alpha diversity box plot of both infected and non-infected groups.

Figure 11: Alpha-facet box bar of evenness, richness, and Shannon diversity.

Figure 12: Principal coordinate analysis (PCoA) of the plot with Bray-Curtis dissimilarity.

Figure 13: Dirichlet multinomial machine learning by clusters of microbial community density.

Figure 14: STAMP differential analysis showing abundance at the genus level.

Figure 15: Networking and proportion of nodes (OTUs color-colored) per phylum found in each cluster of microbial taxonomic composition between infected and non-infected groups.

Figure 16: LDA effect size (LEfSe) analysis of differences in skin microbial abundances between the two groups of infected and non-infected.

Figure 17: Alpha_cowplot diversity of patients' skin microbiota by isolated microorganism in the Surgical Site Infection.

Figure 18: Relative abundancy of patients' skin microbiota with SSI-isolated microorganisms.

S1 File: Ethical clearance of the DESTINE Study at all levels in Uganda.

S2 File: Authors' contribution, list of abbreviations, list and description of tables, figures, and supportive information files.

S3 File: PLOS_One Human Subjects Research Checklist.
