# Supplementary material for: Metagenomic sequencing of the skin microbiota of the scalp predicting the risk of surgical site infections following surgery of traumatic brain injury in sub-Saharan Africa
Source: PLoS One. 2024 Jul 24;19(7):e0303483. doi: 10.1371/journal.pone.0303483 (PMC11268656; doi:10.1371/journal.pone.0303483)
Supplement: S3 File — (PDF) [file pone.0303483.s003.pdf]

Human Participants Research Checklist

Complete the following if your study involved human participants or human participants' data. These questions should be addressed for prospective and retrospective studies.

1. Did you obtain ethics approval for this study?

    - If yes, please upload (file type "Other") the original approval document you received from your ethics committee. <u>If the original document is in another language, please also provide an English translation.</u>

    _YES-- Uploaded

2. If you prospectively recruited human participants for the study – for example, you conducted a clinical trial, distributed questionnaires, or obtained tissues, data or samples for the purposes of this study, please report in the Methods:

    i. the day, month and year of the **start and end** of the recruitment period for this study.

    ii. whether participants provided informed consent, and if so, what type was obtained (for instance, written or verbal, and if verbal, how it was documented and witnessed). If your study included minors, state whether you obtained consent from parents or guardians. If the need for consent was waived by the ethics committee, please include this information.

    YES___ Completed

3. If you are reporting a retrospective study of medical records or archived samples, please report in the Methods section:

    i. the day, month and year when the data were accessed for research purposes

    ii. whether authors had access to information that could identify individual participants during or after data collection

    N/A (This is not a retrospective study).
