# Supplementary material for: Metagenomic sequencing of the skin microbiota of the scalp predicting the risk of surgical site infections following surgery of traumatic brain injury in sub-Saharan Africa
Source: PLoS One. 2024 Jul 24;19(7):e0303483. doi: 10.1371/journal.pone.0303483 (PMC11268656; doi:10.1371/journal.pone.0303483)
Supplement: Attachment — Submitted filename: Point-by-point response 2.pdf [file pone.0303483.s006.pdf]

Dear Editor,

Dear Reviewers,

Thank you very much for the peer-review feedback. Below, we are addressing the additional Editorial and Reviewers' comments as requested. Please find the point-by-point responses to your queries in this summary table:

Q	Query	Response	Line/ Page
**Main manuscript text**			
1.	Page 3, Line 51-52: Frequently, the infection originates from the surrounding residual bacteria of the skin of the scalp where the surgical incision is made [5, 6].<br>**Comment 1:** Authors responded to the comment on original submission that they have modified the text from lines 49-52 by removing the word "scalp" and making the statement applicable to all infections originating from the skin. However, the same is not reflected in the revised manuscript.	**Response 1:** Thank you for your comment. We apologize for having missed deleting the word "scalp". We, therefore, removed it this time.	51-52<br>P.3
2	Page 3-4, Line 66-67: Indeed, in case of any change in the micro-environment of the body, a shifting paradigm of the role of local bacteria to potential microbial infections [10, 11].<br>**Comment 2:** The sentence is still incomplete and requires rephrasing.	**Response 2:** Thank you for this observation. We have deleted the incomplete sentence with its references, and also rephrased the following one to elucidate the gap in the literature as follows:<br>"The skin microbiota of the scalp contains potential contributors to the development of SSI, yet the role	64-68<br>P.3-4

		of its composition in predicting post-operative infection risk in neurosurgery remains poorly understood.”	
3	Page 6, Line 117: Vancomycine<br>**Comment 3:** Correct the typo. Additionally, please write the names of all antibiotics within the sentence in lower case letters only (including the first letter).	**Response 3:**<br>Thank you. This has been corrected. The names of all the antibiotics within the sentence are now written in lowercase letters.	115-117<br>P.6
4	Page 6, Line 120-121: Postoperatively, they also received additional intravenous antibiotherapy in continuation or adjustment in case of evidence of infection........<br>**Comment 4:** The above statement raises concern and require further clarification. What do these words 'continuation or adjustment' refer to? Does it mean that antibiotics were continued even after the surgical intervention (antIbiotic prophylaxis) in all patients, irrespective of the risk or, occurrence of SSI?<br>PLEASE CLARIFY<br>Also, replace 'antibiotherapy' with 'antibiotic treatment'.	**Response 4:**<br>Thank you for your concern.<br>-Regarding the continuation of antibiotics treatment (the so-called “antibiotics cover”) after prophylaxis antibiotics in our center, and also in many other centers, it is common practice in many for neurotrauma surgery, to consider the surgical wound from head injury as class II or III wound (clean-contaminated or contaminated wound by presumption); so this is the rationale of continuation of antibiotics therapy since there is a higher risk of potential SSI due to scalp contusion, scalp wound, bone fracture, dural tear, brain contusion, CSF leakage, and even from remote sources from other body contused	117-118<br>P.6

		tissues as it is the case with patients with history of road traffic accident. Therefore, the benefit of antibiotic treatment following prophylaxis is relatively higher.<br><br>-Regarding the adjustment: In the case of the development of SSI, and the availability of antibiotic susceptibility patterns, it is a common practice to shift the antibiotic treatment based on the drugs' susceptibility.<br><br>-Antibiotherapy was replaced by antibiotics treatment.	
5	Page 7, Line 135: diagnosis<br>**Comment 5:** Add the word 'clinical' before 'diagnosis'.	**Response 5:**<br>The word …"clinical" diagnosis… was adjusted.	134<br>P.7
6	Page 8, Line 196: cutadapt<br>**Comment 6:** 'cutadept' to be written as 'Cutadept'.	**Response 6:**<br>Cutadapt was adjusted as requested.	195<br>P.8
7	Page 16, Line 322: An igragh.degree<br>**Comment 7:** Probable typo	**Response7:**<br>This is not a typo, but the correct word**.**<br>An "**igraph.degree**" is a function in the igraph library (within the R packages) which analyzes and visualizes the strength of a network between microbial communities.<br>No change has been made.	320<br>P.16

8	Page 20, Line 411-414: TBI Patients who undergo emergency neurosurgical intervention may take several hours to days without adequate incisional site preparation on the skin, and again this is worsened by a complex environment of the densely hairy region of the head.<br>**Comment 8:** The intent of the above sentence is still not clear. Why or for what TBI patients may take several hours? Please specify.	**Response 8:** In the context of sub-Saharan Africa (SSA), even in the High-income country setting, TBI patients spend several hours being transferred from the injury site to the primary hospital, then referred to the neurosurgery center, then obtaining a brain CT scan, theatre space, etc. This time can be longer in the context of a weak referral system as is the case in SSA.<br>We have added a statement to improve the sentence following your comment:<br>"TBI Patients who undergo emergency neurosurgical intervention in SSA may take several hours to days (referral to neurosurgery, brain CT scan, theatre space) without adequate incisional site preparation on the skin, and again this is worsened by a complex environment of the densely hairy region of the head."	410-411<br>P.20
**Tables**			
9	Table 1:<br>**Comment 9:** There discrepancy in the absolute numbers and percentage calculation for different genders in the table is yet not sorted in the revised manuscript. Please note that as per the	**Response 9:**<br>Thank you for your detailed observation. We realized revised this discrepancy, and corrected it from the data base.	Table 1

	statistics in the table, there were 2 Females and 9 Males (totalling to 11 patients) under the SSI (Yes) group. But the actual number of patients in SSI(Yes) group was 12.<br>Similarly, there were 4 Females and 42 Males (totalling to 46 patients) under the SSI (No) group. But the actual number of patients in SSI(Yes) group was 45.<br>Please make correction, and do re-calculation of percentages and p-value.	We also did the calculation and revise the Fisher Exact p-value as requested regarding the variable "Sex" of participants.<br><br>We also realized that the previously corrected percentage in the total was not relevant, thus, we recovered the row percentage as presented initially.	
10	**Comment 10:**<br>Also, in table 1, the statistical analysis done and mentioned for 'Interval between injury & skin swab collection' does not suit. Fisher's exact test mentioned for the mean (+SD) days does not seem appropriate.<br>Authors may mention this newly added information only in the text if it is not exactly fitting in Table 1.	**Response 10:**<br>Thank you for this comment; we have edited Table 1 to match and fit this information by specifying the appropriate type of test and p-value (Mann-Whitney and Fisher).	Table 1
11	Table 2:<br>**Comment 11:**<br>Please split or hyphenate 'piperacillintazobactam' which is still mentioned as a single word in the row for patient serial no. 10.	**Response 11:**<br>We have split it with a hyphen as requested 'Piperacillin-Tazobactam'.	Table 2

Best regards,

Corresponding Author
