# Supplementary material for: Metagenomic sequencing of the skin microbiota of the scalp predicting the risk of surgical site infections following surgery of traumatic brain injury in sub-Saharan Africa
Source: PLoS One. 2024 Jul 24;19(7):e0303483. doi: 10.1371/journal.pone.0303483 (PMC11268656; doi:10.1371/journal.pone.0303483)
Supplement: Attachment — Submitted filename: Point-by-point responses to the Editor_Reviewers.docx [file pone.0303483.s005.docx]

Dear Editor,

Dear Reviewers,

Thank you very much for the peer-review feedback. I salute the detailed review and comments on our manuscript, and mostly the final inputs that we have brought have improved the overall quality of the manuscript.

Below, we are addressing the additional Editorial and Reviewers’ comments as requested by the journal guidelines. Please find the point-by-point responses to your queries in this summary table:

| Q | Query | Response | Line/  Page |
| --- | --- | --- | --- |
|  | **INTRODUCTION** |  |  |
| 1. | Lines 45-46: One of the post-operative challenges of the surgical management of traumatic brain injury (TBI) is the occurrence of surgical site infections (SSI) in sub-Saharan Africa (SSA) [1-3].  **Comment:** None of the three cited references actually mentioned the study findings of sub-Saharan Africa.  Please replace it with more suitable references. | **Response 1:** We have removed those references, and provided updated and more suitable ones from sub-Saharan Africa. | 45-46  P.3 |
| 2 | Lines 47-49: This post-infectious complication is the commonest morbidity that leads to subsequent postoperative mortality among TBI patients up to 3 months after the initial surgery, with a prolonged hospitalization stay, increased healthcare costs, and impaired patient outcomes [4].  **Comment:** The cited reference is almost four decades old. Please cite 2 more suitable and latest references. | **Response 2:** We have removed that old reference, and cited a more suitable and latest one. | 47-49  P.3 |
| 3 | Lines 49-52: The bacterial origin of the infection during surgical procedures is very complex. They can be endogenous, exogenous (contamination), or both. Frequently, the infection originates from the surrounding residual bacteria of the skin of the scalp where the surgical incision is made [5-7].  **Comment:** The cited reference number 5 mentions specifically mentioned about SSI after cesarean section. The other two studies did not mention anything on SSI. Please cite more suitable references or modify the text without specifying scalp SSI. | **Response 3:** Thank you for your critical observation of the references and citing.  We have modified the text from lines 49-52 by removing the word "scalp" and making the statement applicable to all infections originating from the skin. However, we have removed the previous reference [6] on the bacterial biofilms. | 49-52  P.3 |
| 4 | Line 64: …….in normal circumstances…..  **Comment:** The above part of the sentence seems redundant and misleading since biofilm formation may not be considered normal. | **Response 4:** Thank you for your comment. This was addressed in specific line of the manuscript by removing the potentially misleading and redundant word biofilm formation, and its reference. | 67  P.4 |
| 5 | Lines 65-67: In case of any change in the micro-environment of the body, a shifting paradigm of the role of local bacteria to common microbial infections up to 65% [6, 10].  **Comment:** The above sentence is misleading. The cited reference number 10 actually mentioned that biofilms may be responsible for up to 65% of all infections. | **Response 4:** We have changed the misleading sentence and reference about the biofilms, and rewrote the sentence as requested with new references. | 67  P.4 |
| 6 | Lines 77-78: 16S RNA sequencing  **Comment:** Please mention ‘16S rRNA sequencing’. | **Response 6:** This was corrected as suggested. | 78  P.4 |
| 7 | Lines 84-88: The uniqueness of this research…… is more prone or protective against skin infections.  **Comment:** It appears that the authors tried to justify their study findings, not only the need for the study in the above sentences. Therefore, these lines should better be suited as part of the DISCUSSION and may be moved to the latter part of the manuscript. | **Response 7**: These sentences were moved to the discussion section as requested | 364-368  P.18 |
|  | **MATERIALS AND METHODS** |  |  |
| 9 | Line 94: DESTINE-study  **Comment:** Please mention any national or international registry platform where the study may have been registered and mention the registration number. | **Response 9:** The study is registered in the Uganda National Council of Science and Technology (UNCST) with the number HS1284ES. We have included the registration number in the manuscript. | 90-91  P.5 |
| 10 | Lines 95-102: This present study…………………steroid treatment, or with comorbidities.  **Comment:** Two subheadings, ‘Inclusion’ and ‘Exclusion’ criteria, may be created for this paragraph for better understanding of readers. | **Response 10:** the sub-headings “Inclusion” and “Exclusion criteria” have been created under the new paragraph of the heading “study participants.” | 94-103  P.5 |
| 11 | Line 99:…hemodynamically stable, and whose informed written consent was obtained…  **Comment:** Given the serious nature of the patients, please clarify whether written consent was only sought from patients or consent from patients’ next to kin was considered (in case patient is unable to provide consent. | **Response 11:** We stated these details in the ethical section in the later paragraphs; we added an additional comment: "… obtained by themselves or by their legal next-of-kin.” | 98  P.5 |
| 12 | Lines 103-104: They all received………….the team readiness  **Comment:** This is a crucial part and needs to be elaborated. Please mention the types/names of antibiotics used for prophylaxis. How long was the prophylaxis continued? These information is crucial because they have a direct effect on the clinical outcomes being considered in the present study. | **Response 12:** Thank you for this comment. We have added the types and dosage of the antibiotic therapy as requested.  In fact, this point is very crucial but we initially removed it due to the focus on the metagenomics data. | 114-122  P.6 |
| 13 | Line 109: abundancy  **Comment:** Please replace the above word with ‘abundance’. Also make similar changes elsewhere in the manuscript. | **Response 13:** Thank you for this observation.  We also attempted initially to write it as abundance during manuscript preparation, but the current correct metagenomics metric is written as "**Abundancy**” in English.  We are afraid to make this change as requested.  We also argued in the team that most dictionaries do not recognize the word "Abundancy" by suggesting "Abundance", but unfortunately the correct terminology used in the metagenomics metrics remains still "abundancy". | N/A |
| 14 | Lines 113-114: Collection of the skin swab: During the perioperative period, just after obtaining informed consent, a skin swab of the surrounding normal skin.  **Comment:** It is imperative to mention the duration of stay in the hospital prior to the collection of skin swabs during the peri-operative period. This is particularly important since a prolonged stay in the hospital may adversely affect scalp colonization by organisms from the hospital environment. | **Response 14:** Thank you for your observation. This is a very critical information that we have added now. Since you mentioned the time between admission and sampling, we have even chosen to include more interesting information that is from the timing of injury to sample collection so that we may have a clearer idea when attempting to reproduce the result of this work in the future.  Indeed, we have included this information in the result section of the manuscript body, as well as in Table 1 “interval of time between injury and skin swab collection expressed in days”. | 209  P.9  &  225  P.11 |
| 15 | Line 114: e.g: retro-auricular skin at the hairline  **Comment:** Is there any specific reason for choosing the retro-auricular skin? Please clarify | **Response 15:** We chose the retro-auricular region because it is less in contact with the hospital environment like bed linens, and others, especially for patients lying supine on the bed for several hours. We also include this comment in the methods section justifying this site. | 156-158  P.7-8 |
| 16 | Line 121-134: DNA extraction………..16s rRNA sequencing  **Comment:** This entire paragraph may not be required given the length of the manuscript. Instead, the authors may choose to mention that DNA extraction was carried out following the manufacturer’s recommendation. | **Response 16:** The entire paragraph was deleted, and a statement of the manufacturer’s recommendation has been mentioned instead. | 163-164  P.8 |
| 17 | Line 138: forward and reverse primers  **Comment:** Please mention sequences of all forward or reverse primers or upload them as supplementary files. | **Response 17:** the sequence of the forward or reverse primers was added in the manuscript:  Target -16s V3V4  16S Amplicon PCR Forward Primer = 5' TCGTCGGCAGCGTCAGATGTGTATAAGAGACAGCCTACGGGNGGCWGCAG3'  16S Amplicon PCR Reverse Primer = 5' GTCTCGTGGGCTCGGAGATGTGTATAAGAGACAGGACTACHVGGGTATCTAATCC 3' | 168-172  P.8 |
| 18 | Line 154: Illumina platform  **Comment:** Please mention the exact name of the instrument with the model number, manufacturer and country of origin. | **Response 18:** We have mentioned the exact name with the model number and country of origin in the main manuscript:  the Illumina MiSeq, sequencing machine (California, USA) model# M02903, serial number 410-1003. | 187-189  P.9 |
| 19 | Lines 156-159: After sequencing………………….microbial communities present in the samples.  **Comment:** Please mention all software names with their specific use in the bioinformatic analysis. | **Response 19**: We have mentioned the softwares and their specific use in the bioinformatics analysis. | 192-239  P.9-11 |
| 20 | Lines 160-165: Bioinformatics  **Comment:** Please mention all software names with their specific use in the bioinformatic analysis. It is also desirable to include the URL link of the applications within parentheses after the names of the software. | **Response 20**: We have mentioned all the software and their specific use in the bioinformatics analysis. We also included the URL link of the applications in parentheses after the softwares. | 187-236  P.9-11 |
|  | **RESULTS** |  |  |
| 21 | Lines 195-196: Indeed, in addition to the study inclusion criteria, we convened with those 57 patients because their samples were amplifiable with a successful quality check for sequencing.  **Comment:** The above sentence raises some concerns. Does it actually mean that non-ampilfiablility and/or failure in quality checks are also criteria for exclusion? If so, this should be mentioned as an Exclusion criterion under MATERIALS NAD METHODS. | **Response 21:** Thank you for this comment. Based on the study design, we could have not taken into account the amplifiability and quality check failure of samples initially, which is why we convened on these 57 samples. As suggested, we have added this as part of the exclusion criteria under the methods section. | 102-103  P.5 |
| 22 | Lines 204-206: The samples of the 12 patients………………. antibiotic susceptibility  **Comment:** This investigation is very important and has been totally omitted from the MATERIALS AND METHODS. Please mention it under a separate subheading, ‘Laboratory investigation for diagnosis of SSI’ under materials and methods. Mention the type of antibiotic susceptibility testing performed and the clinical breakpoints to denote susceptible, intermediate, and resistant. | **Response 22:** Thank you very much for this comment. We have enriched the methods section by adding the omitted important information. Please find the sub-section of study procedures divided now into 2:  In study procedure I (Clinical management and bacteriological studies), we have created a sub-section of the “clinical diagnosis of the surgical site infection” with details, and also the subsection of the “laboratory investigations for microbiological culture identification assays and drug susceptibility testing following the diagnosis of surgical site infection”, where the requested comments were incorporated, as well as the antibiotics susceptibility. | 134-151  P.7 |
| 23 | Lines 206-209: We found that mono-infection……….12 patients with SSI.  **Comment:** Please rephrase the above sentence for clarity.  Replace 'mono-infection' with 'mono-microbial infection'. Please make similar changes elsewhere in the manuscript.  Please mention both Genus and species names for the isolates. | **Response 23:** Thank you for this correction. We have replaced mono-infection with mono-bacterial infection elsewhere.  We also mentioned both Genus and species of the cultured micro-organisms (*Staphylococcus aureus 2 + Enterococcus spp* 4). | 264-265  P.12 |
| 24 | Lines 209-210: One case of mortality due to intracranial SSI………………… associated with Escherichia Coli and Klebsiella pneumoniae.  **Comment:** Please mention ‘intracranial infection’ in place of ‘intracranial SSI’. Please write species names in lowercase letters. All genera and species need to be italicized. Check all organisms’ names in the manuscript. | **Response 24:** We have replaced intracranial SSI with Intracranial infection as requested.  We also wrote specific names in lowercase, as well as all genera and species italicized throughout the manuscript. | 268  P.12 |
| 25 | Lines 218-220: The phyla of………………………. proportion at adult age.  **Comment:** It is not clear which age group is considered as the pediatric population in the study. As per Figure 4, it seems to be that there were three patients in the pediatric group and two patients in the adult group. Please clarify. | **Response 25:** Thank you for your observation. We initially added a text of the description of the figure outside Figure 4.  To make it less confusing, we have added the legend to the bottom of Figure 4 (now Figure 3). We also updated the information on the manuscript body.  We included a legend as follows:  P1 = Pediatric group: < 5 years  P2 = Pediatric group: ≥ 5 - 11 years  P3 = Pediatric group: ≥ 12 - 17 years  A1 = Adult group: ≥ 18 - 39 years  A2 = Adult group: ≥ 40 years | 276-279  P.14 |
| 26 | Lines 238-243: Alpha diversity was measured by……………….. of infected and non-infected.  **Comment:** I think that the information mentioned in this paragraph is better suited under the Statistical Analysis under MATERIALS AND METHODS. | **Response 26:** Thank you for your information. We have transferred and merged this initial portion of this paragraph into the statistical heading of the Methods section.  The remaining are still part of the results since showing the comparison of microbial communities as study findings. | 214-217  P.10 |
| 27 | Line 241: Wilcoxon test  **Comment:** Please clarify which variant of Wilcoxon test was used. | **Response 27:** Thank you again, we added clarification, and used the Wilcoxon rank-sum test. | 298  P.15 |
| 28 | Line 259: Ochnobacterium  **Comment:** Probable typo. | **Response 28:** Correction was made to Ochnobactrum | 316  P.16 |
| 29 | Lines 263-264: Co-occurrence network graphs of the skin microbiota and their node taxonomic composition between the 2 groups of interest.  **Comment:** The above sentence is incomplete or grammatically incorrect. | **Response 29:** We have rephrased this sentence, and made it grammatically correct. | 320-326  P.16 |
| 30 | Lines 269-270: LDA effect size (LEfSe) analysis of between two-group differences in skin microbial abundances for personality traits.  **Comment:** The above sentence is incomplete or grammatically incorrect. | **Response 30:** We have rephrased this sentence, and made it grammatically correct. | 328-331  P.17 |
| 31 | **DISCUSSION**  The sub-headings under discussion are not required and should be deleted. | **Response 31:** All those sub-headings were removed from the discussion section as requested. | N/A |
| 32 | Lines 284-285: We found a higher antimicrobial resistance to common antibiotics, and it is well known from microbiologic studies using the biofilm of the bacterial communities [6, 14].  **Comment:** The above sentence is misleading and needs to be rephrased for a better understanding of the readers. Please ensure that both the references mentioned within brackets have been cited appropriately. | **Response 32:** Thank you for this comment.  We have rephrased the misleading sentences and also deleted the less appropriate references. We have added new references. | 344-346  P.17 |
| 33 | Line 294: ……….. the 3rd and 4th abundant genera…..  **Comment:** Please mention ‘respectively’ after ‘genera’. | **Response 33:** Thank you.  This was done accordingly. We have added the word respectively after genera. | 355  P.18 |
| 34 | Lines 321-322: Patients who did not develop showed a more balanced and stable microbiome like in the general skin microbiome as described by Egert et al……  **Comment:** The above part of the sentence is incomplete or grammatically incorrect. | **Response 34:** Thank you again.  We have rephrased this sentence, and made it grammatically correct by adding: “... Who did not develop **SSI** showed…” | 380-382  P.19 |
| 35 | Line 349: multi-resistant  **Comment:** Replace ‘multi-resistant’ with ‘multidrug-resistant’. | **Response 35:** Thank you, this was done as requested. We replaced multi-resistant with multidrug-resistant. | 408  P.20 |
| 36 | Lines 352-353: TBI Patients who undergo emergency surgery may take several hours to days without adequate skin preparation due to the presence of the hair.  **Comment:** The above sentence seems incomplete. Please check the sentence and rewrite it for a clear understanding. | **Response 36:** We have rephrased this sentence, and made it grammatically correct.  We rephrase it as: “TBI Patients who undergo emergency neurosurgical intervention may take several hours to days without adequate incisional site preparation on the skin, and again this is worsened by a complex environment of the densely hairy region of the head.” | 411-414  P.20 |
| 37 | Lines 392-393: List of abbreviations  **Comment:** ‘OUT’ should be written as ‘OUT’. | **Response 37:** Thank you for this observation; correction has been made as requested (OUT to OTU) | 467  P.23 |
|  | **COMMENTS ON TABLES** |  |  |
| 38 | **Table 1**  There is a major discrepancy in the absolute numbers and percentage calculation for different genders. The column totals of males and females do not match the total number of patients developing SSI and patients without SSI. Accordingly, the Fisher's exact test p-value needs to be recalculated. | **Response 38:** Thank you for your observation; in fact, initially we used both row percentages for the sub-group, and column percentages for the total.  We have harmonized the % to solve the discrepancy as suggested, and the total % is 100%.  Fisher's exact test in the tables was recalculated and updated. | Table 1 |
| 39 | **Table 2:**  In serial number 10, piperacillin-tazobactam has been written as a single word. Authors may also choose to mention cotrimoxazole as ‘trimethoprim-sulfamethoxazole’. | **Response 39:** Thank you for your observation. Corrections have been made as suggested. | Table 2 |
|  | **COMMENTS ON FIGURES** |  |  |
| 40 | A large number of figures are included in the manuscripts. Therefore, trivial figures like Figure 3 may be deleted. What is P1, P2, P3, A1, A2 in Figure 4? | **Response 40:** The initial figure 3 was deleted, and the subsequent picture became figure 4 became 3 in the order of appearance in the manuscript, and so on.  In the Original Figure 4: we included a legend as follows:  P1 = Pediatric group: < 5 years  P2 = Pediatric group: ≥ 5 - 11 years  P3 = Pediatric group: ≥ 12 - 17 years  A1 = Adult group: ≥ 18 - 39 years  A2 = Adult group: ≥ 40 years | Figure 3 |
|  | **REVIEW COMMENTS TO THE AUTHOR** |  |  |
| 41 | **Reviewer #1:**  The study outlined presents an investigation into the relationship between scalp microbiota composition and the risk of Surgical Site Infections (SSIs) following Traumatic Brain Injury (TBI) surgery. While the study provides valuable insights, there are few lacunae where further clarification or improvement is needed: | Thank you for your honest summary feedback.  We have provided clarification and made corrections to the above-mentioned concerns. | N/A |
| 42 | 1. Environmental contamination: The possibility of contamination with environmental flora is not considered, especially when the target population are admitted after potential RTC which may introduce bias into the scalp skin microbiota composition and their representativeness of scalp microbiota diversity. Additionally, the choice of cleaning the sampling sites to get rid of sand or mud, etc. should be addressed. | Thank you for this observation. We have added these comments on limitations to address your concern that we totally find legit. As mentioned in our protocol, we first cleaned the site with normal saline and sterile gauze to remove (better to say significantly reduce) the superficial contaminations (sands, etc.). The skin microbiome is normally embedded under the superficial layers of the epidermis, and may not be affected by gentile rubbing with sterile gauze with neutral cleaning sterile water (normal saline) without an antibacterial solution. | 440-448  P.22 |
| 43 | 2. Sample Size and Generalizability: The sample size of the study is not explicitly justified or powered. Given the complexity of microbiota analysis and the potential variability in SSI occurrences, a larger sample size would enhance the study's statistical power and generalizability of the findings. The study acknowledges its limitation of a relatively small sample size from a single center. This raises concerns about the generalizability of the findings to broader populations. Larger multi-center studies would provide more robust evidence. | Thank you for reiterating our limitations. As you can see, there were some difficult logistical limitations to achieve a much larger sample size, such as:  - The patients’ selection had cut down the number in recruitment (relatively stable patients, no signs of infections, etc…);  - the relatively rare incidence of surgical site infection with clinic-radiological and bacteriological evidence after surgery of head trauma within a 1 year of recruitment combined with a 3-month follow-up for each patient;  - The limited research cost for the expensive metagenomics sequencing in Uganda could not allow us to accommodate many more samples.  We hope our study will serve as a baseline for a much more robust large-scale multi-center prospective study to confirm our hypothesis with stronger evidence. | N/A |
| 44 | 3. Causal Inference: While the study suggests an association between scalp microbiota dysbiosis and SSI risk, it does not establish causality. Further mechanistic studies are needed to elucidate the underlying biological mechanisms driving this association.  Addressing these lacunae would strengthen the study's design, rigor, and relevance, enhancing the reliability and significance of its findings. | Thank you for reflecting on our conclusions. As we said, although we had a predicting risk measurement based on our prospective design, we reported mainly the association instead of causal inference. The main reason is that the occurrence of surgical site infection is multi-factorial, so dysbiosis could just be one of the pathways in the pathological process.  As suggested, we have indeed addressed these lacunae in the paragraph before the limitations, and also in the conclusion | P.22 |
| 45 | Overall, based on the information provided, the manuscript appears to be technically sound, and the data support the conclusions drawn by the researchers. | Thank you. | N/A |
| 46 | **Reviewer #2:**  The manuscript is well written. The authors have thoroughly researched the impact of scalp microflora on the infections following traumatic brain injury. The manuscript can be accepted in the current form. | Thank you for your feedback. | N/A |

| 47 | While revising your submission, please upload your figure files to the Preflight Analysis and Conversion Engine (PACE) digital diagnostic tool, <https://pacev2.apexcovantage.com/>. PACE helps ensure that figures meet PLOS requirements. To use PACE, you must first register as a user. Registration is free. | This was done and all the figures passed the digital diagnosis through PACE as recommended, and automatic conversion of pictures to TIF format was done. | N/A |
| --- | --- | --- | --- |

Best regards,

Corresponding Author
